# Feasibility study of Bismuth Subsalicylate (BSS) as an addition to standard of care for COVID-19 therapy^☆^

**DOI:** 10.1016/j.curtheres.2022.100667

**Published:** 2022-03-30

**Authors:** Mary Beth Yacyshyn, James Collins, Michelle Chua, Angela Siegwald, Sara Yacyshyn, Valerie Briones-Pryor, Bruce Yacyshyn

**Affiliations:** a Musidora Biotechnology LLC, Cincinnati, Ohio; b Department of Microbiology & Immunology, University of Louisville, Louisville, Kentucky; c Division of Gastroenterology, Hepatology, and Nutrition, University of Louisville School of Medicine, Louisville, Kentucky; d Digestive Diseases & Surgery Institute, Cleveland Clinic, Cleveland, Ohio; e University of Louisville Health, Louisville, Kentucky; f Medpace Inc, Cincinnati, Ohio

**Keywords:** SARS-CoV-2, Bismuth subsalicylate (BSS), helicase

Dear Dr. Walson:

A combination of vaccines and antiviral drugs is needed to fight SARS-CoV-2. Although development and efficacy of SARS-CoV-2 vaccines has been timely, utilizing therapeutic antiviral medications, either repurposed or newly generated, will be key. New oral antiviral medications have been shown to lower hospitalization rates and deaths.^1^^,^^2^ Several repurposed drugs are now being studied with Phase II/IIIB clinical trials.^3^, ^4^, ^5^

We assessed feasibility and tolerability of bismuth subsalicylate (BSS) tablets (Pepto-Bismol; Procter & Gamble, Cincinnati, Ohio) as a 3-day addition to current standard-of-care treatment for mild-to-moderate SARS-CoV-2 at 1 clinical site. BSS has been shown to have antibacterial and antiviral activity.^6^^,^^7^ It has been incorporated into medications used for gastrointestinal indications and has been shown to influence SARS-CoV-2 replication, specifically its helicase.^6^, ^7^, ^8^, ^9^, ^10^ This communication provides preliminary data on the clinical feasibility, acceptability of dosing, outcome measures, and staff/patient participation obtained from the initial open-label portion (10 patients) of clinical trial NCT04811339.

Recruitment, retention of patients, and completion of the initial open-label portion were difficult. Patients were enrolled October 2020 through February 2021. Staff shortages due to absence, infection exposure, fatigue, and generalized COVID-19 fear influenced recruitment as well as the required staff/patient connections and sample collections. Forty-four patients were consented, 3 outpatients and 41 inpatients from the COVID-19 floor of the University of Louisville Hospital. Forty-three patients were unvaccinated. Vaccine availability for nonhealth-care workers and the general Kentuckian population younger than age 70 years began on March 22, 2021. Twenty-five patients did not receive the study drug and were excluded due to medication or negative salivary SARS-CoV-2 test, or withdrew due to transportation difficulties or change of mind. Fifteen of these 25 patients only had telephone contact with a coordinator.

Nineteen patients received the study medication. Ten completed the 3-day open-label study, with no reports of an adverse event. Of the 9 who did not complete the study, 3 reported an adverse event (specifically, bloating and abdominal discomfort), 5 did not continue BSS after discharge due to transportation/distance issues from hospital affecting saliva/stool collection, and 1 was inconclusive for salivary SARS-CoV-2 throughout the 3 days. Seventeen of 19 patients receiving the study drug had personal contact with coordinator. Going forward, plans to complete sample acquisition within a 3-hour distance radius need to be in place and mandatory in-person coordinator contact needs to be emphasized. One of 44 patients became medically unstable between consent and coordinator telephone contact and was transferred to an intensive care unit.

Completion of 48 BSS tablets was challenging for the patients who completed the trial. Of the first 5 who completed the study, only 1 finished 48 BSS tablets. During January 2021, a protocol amendment was filed to decrease the number of total tablets from 48 to 24. The final 5 patients followed this dosing regimen. Even with this adjustment, the same things influenced full dose completion. Inpatient floor nurses would forget to give BSS tablets and the combination of baseline/day1 visit led to fewer tablets taken the first day. Mandatory daily personal supervision by coordinators with patients and floor nurses needs to be implemented for the randomized placebo-controlled study.

Each day, patients recorded their stool frequency, provided stool and saliva samples, and scored 5 common COVID-19 symptoms: cough, headache, fatigue, and shortness of breath. Patients were asked by the coordinator (telephone or in person) to self-score (from 0 to 3) the 5 symptoms at baseline/day1 (before BSS), after 24 hours/day 2 and 48 hours/day 3. The final salivary testing and symptom scores were taken before the last dose of BSS. The primary objective was to measure diarrhea. However, it became apparent after 1 month that diarrhea was not a typical COVID-19 symptom at our site. Two of 44 consented patients presented with diarrhea and after analysis, stool frequency did not change during the study period. Therefore, after study completion salivary viral clearance (negative reverse-transcription–loop-mediated isothermal amplification test) along with a patient's daily COVID-19 symptom scores became key assessments. Due to patients’ forgetfulness and staff shortage, not all fecal samples were collected. Most limiting was lack of a fourth-day of sample collection or scoring. Going forward, it will be clearly outlined with staff that final samples and scoring should be carried out 24 hours after completion of final BSS dose.

Two outpatients and 8 inpatients completed the study; those with incomplete dosing were all inpatients (see the Table 1). The baseline 5- and 3-symptom patient scores are in the Table 1. Hypertension was the most reported preexisting comorbidity. Pneumonia and/or pleural effusions were the most reported COVID–19-related morbidity. Inpatients took an average of 4 preexisting medicines and were given an average of 6 new medicines (for COVID-19) in hospital. The 2 outpatients with mild disease were younger, had fewer preexisting comorbidities but a higher baseline COVID-19 symptom score (7.5 [1.5] out of 15). At the end of the 3-day open-label BSS study, the mean overall 5-symptom score decreased after 48 hours of taking the study drug (see the Table 1). Cough, headache, and fatigue changed the most during BSS treatment (see the Table 1). Seven of 10 patients resolved (score of 0) cough, 3 out of 4 resolved headache, and 2 out of 7 decreased perceived fatigue.

**Table 1:** Demographic characteristics of patients with mild-to-moderate SARS-CoV-2 treated with bismuth subsalicylate (BSS) tablets (Pepto-Bismol; Procter & Gamble, Cincinnati, Ohio) as a 3-day addition to current standard-of-care treatment.

	Complete BSS (n = 10)	Complete BSS: Inpatient only (n = 8)	Incomplete BSS: Inpatient only (n = 9)	No BSS (n = 25)
Age, y Mean (SEM) Range	52.7 (6.89) 25-78	59.1 (6.83) 27-78	65.2 (2.9) 51-79	60.6 (2.9) 24-84
Body mass index Mean (SEM) Range	30.9 (2.2) 21-44	33.3 (1.9)* 26-44	28.7 (1.6)* 17-33	
Gender Female Male	5 5	4 4	3 6	11 14
Race Black Caucasian, Hispanic Native Pacific Islander Caucasian	2 1 0 7	2 1 0 5	2 0 0 7	7 1 1 16
No. of BSS tablets taken Mean (SEM) Range	27.7 (2.8) 20-48	28.1 (3.3) 20-48	8.1 (2.8) 2-28	NA
No. of preexisting home medicines Mean (SEM) Range	3.8 (1.2) 0-13	4.1 (1.5) 0-13	4.8 (1) 1-10	ND
No. of new hospital medicines for COVID-19 Mean (SEM) Range	NA	5.9 (1.1) 1-10	6.0 (0.61) 3-9	ND
No. of COVID-19 symptoms days before study entry Mean (SEM) Range	9.4 (1.6) 5-19	10.1 (2) 5-19	12.1 (3.0) 6-30	ND
No. of preexisting comorbidities Mean (SEM) Range	3.7 (1) 0-10	4 (1) 0-10	4.7 (0.5) 3-8	ND
No. of COVID-19 morbidities Mean (SEM) Range	3.5 (0.9) 0-9	4.4 (0.9) 1-9	2.4 (0.4) 1-4	ND
Baseline 5-symptom score Mean (SEM) Range	3.8 (0.9) 1-9	2.9 (0.7) 1-6	3.2 (1.3) 0-11	ND
Day 3 5-symptom score Mean (SEM) Range	2.9 (0.8) 0-7	2.7 (1.0) 0-7	ND	ND
Baseline 3-symptom score Mean (SEM) Range	2.4 (0.5) 0-5	2.0 (0.5) 0-4	2.1 (0.9) 0-8	ND
Day 3 3-symptom score Mean (SEM) Range	1.4 (0.6) 0-4	1.5 (0.6) 0-4	ND	ND

ND = no data.

⁎ *P* = 0.0464 based on nonparametric Mann-Whitney test (Prism version 9.3.1; GraphPad Software, San Diego, Calif).

**Figure 1:**
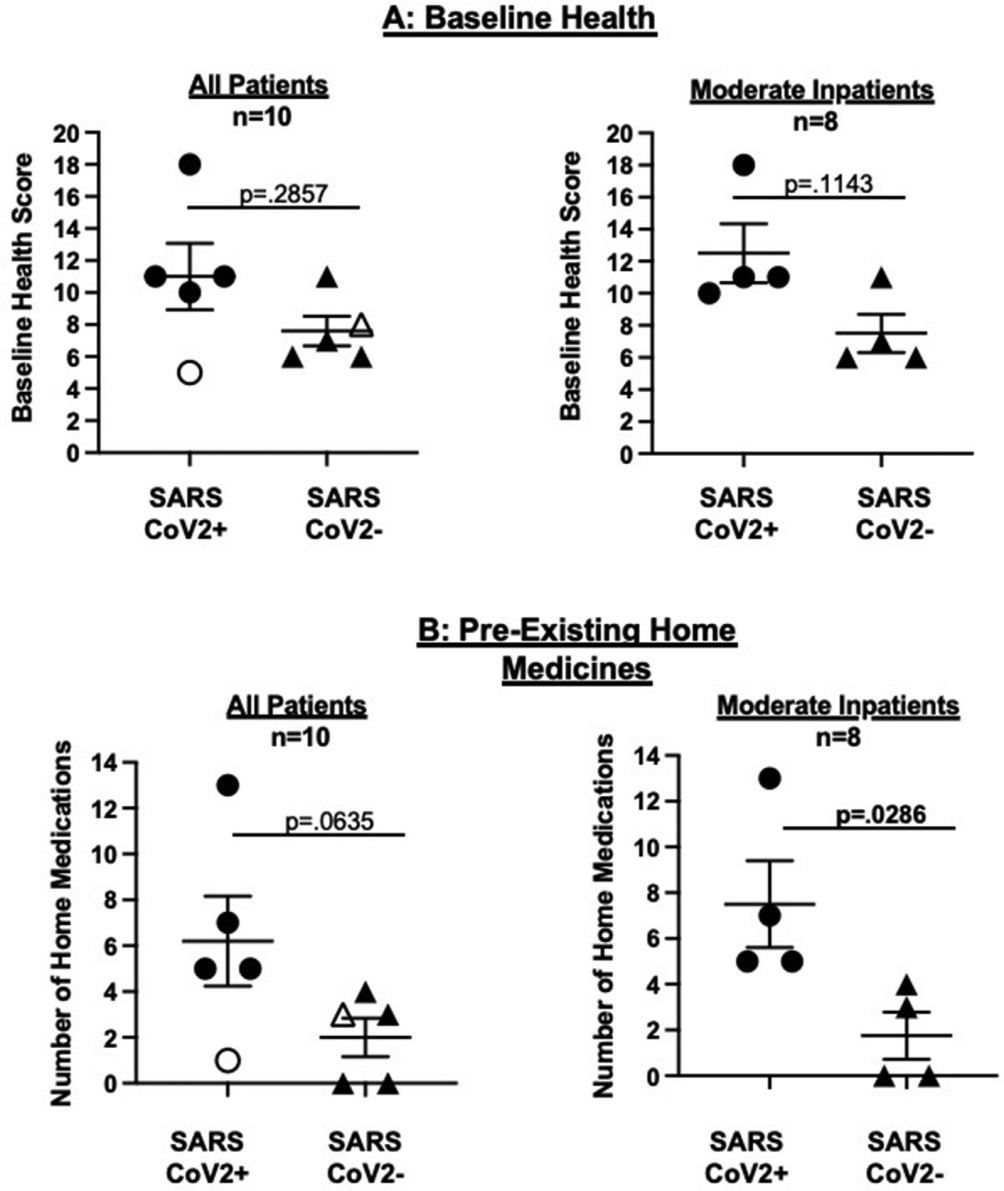
SARS-CoV-2 status on day 3 versus baseline health and medication status. (A) Baseline health scores. Baseline health scores were calculated by using the sum of the number of preexisting comorbidities + the number of baseline COVID–19-related 5 symptoms + number of COVID-19 morbidities. (B) The number of any existing home medications that patients were taking. Open symbols represent outpatients and closed symbols represent inpatients. Statistics were performed using Prism 9 for Mac OS (GraphPad Software, San Diego, California). Nonparametric Mann-Whitney *P* values were reported.

Fifty percent of patients who completed the BSS study became negative for salivary SARS-CoV-2 after 48 hours of BSS. The clearance of SARS-CoV-2 appears to be related to baseline health status and existing home medicines (see the Figure 1). The baseline health status score was derived from the number of preexisting comorbidities + number COVID–19-related morbidities + number of baseline COVID–19-related symptoms. One outpatient took 16 tablets during the first 24 hours, felt better, took 4 BSS tablets on day 2, and remained SARS-CoV-2 positive on day 3. This demonstrates key limitations of the study, the need for oversight by coordinators for dose completion, and final sample collection should be 24 hours after final BSS dose.

Working in the prevaccine environment was challenging; however, we found that over-the counter BSS could influence virus symptoms and salivary SARS-CoV-2 clearance. BSS could be given and tolerated with current standard of care COVID-19 treatments. BSS is inexpensive, easily transportable, stored at room temperature, and has a known safety profile and antiviral properties. This investigation supports further studies of BSS for COVID-19.
